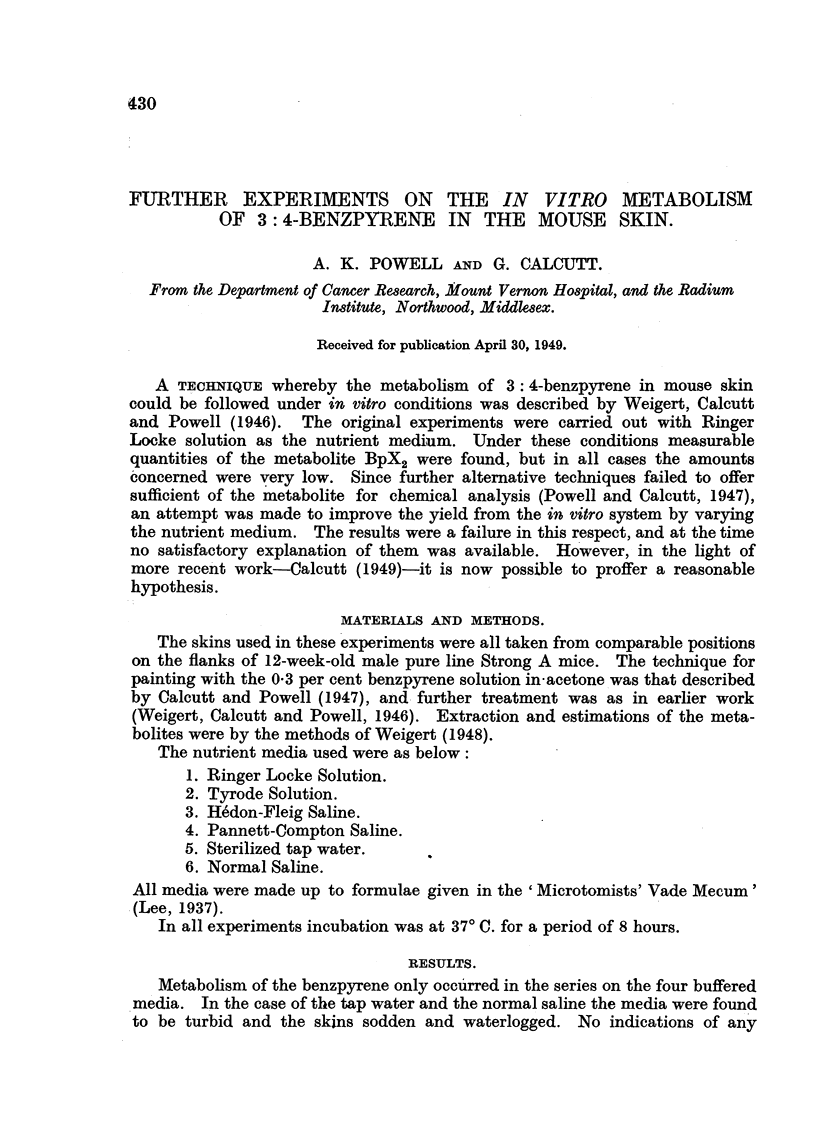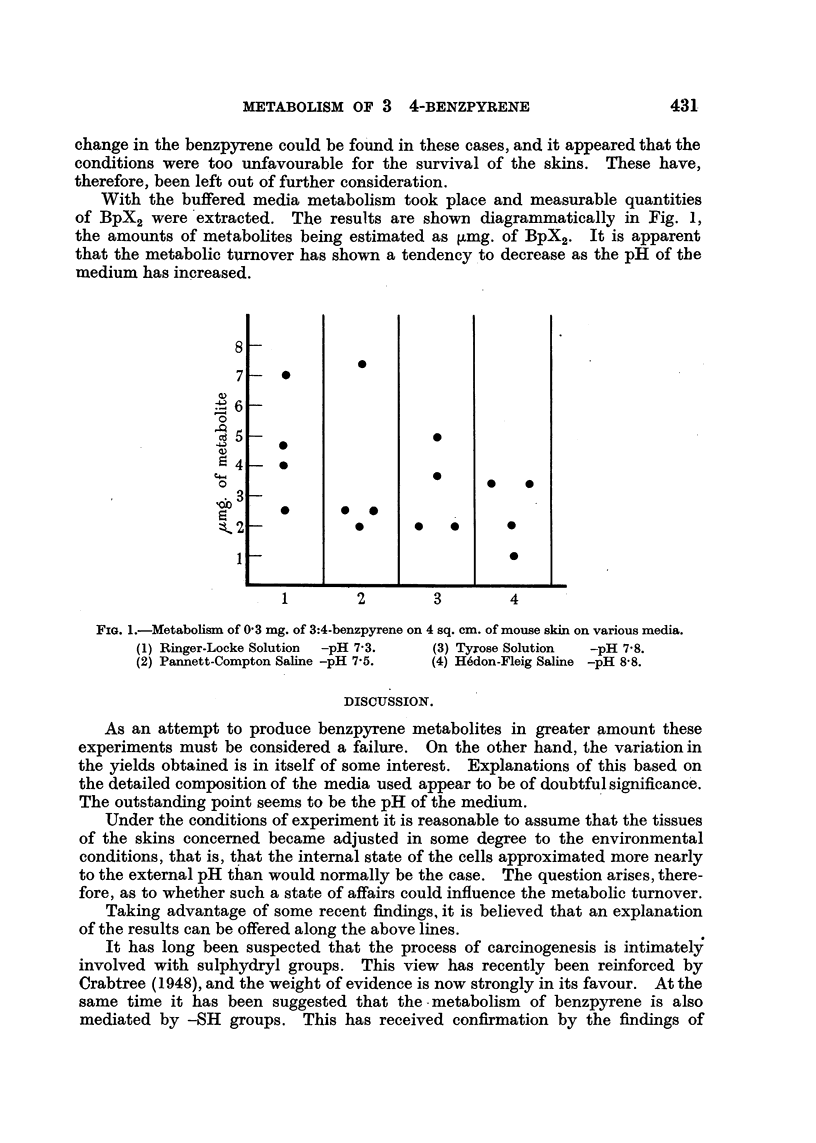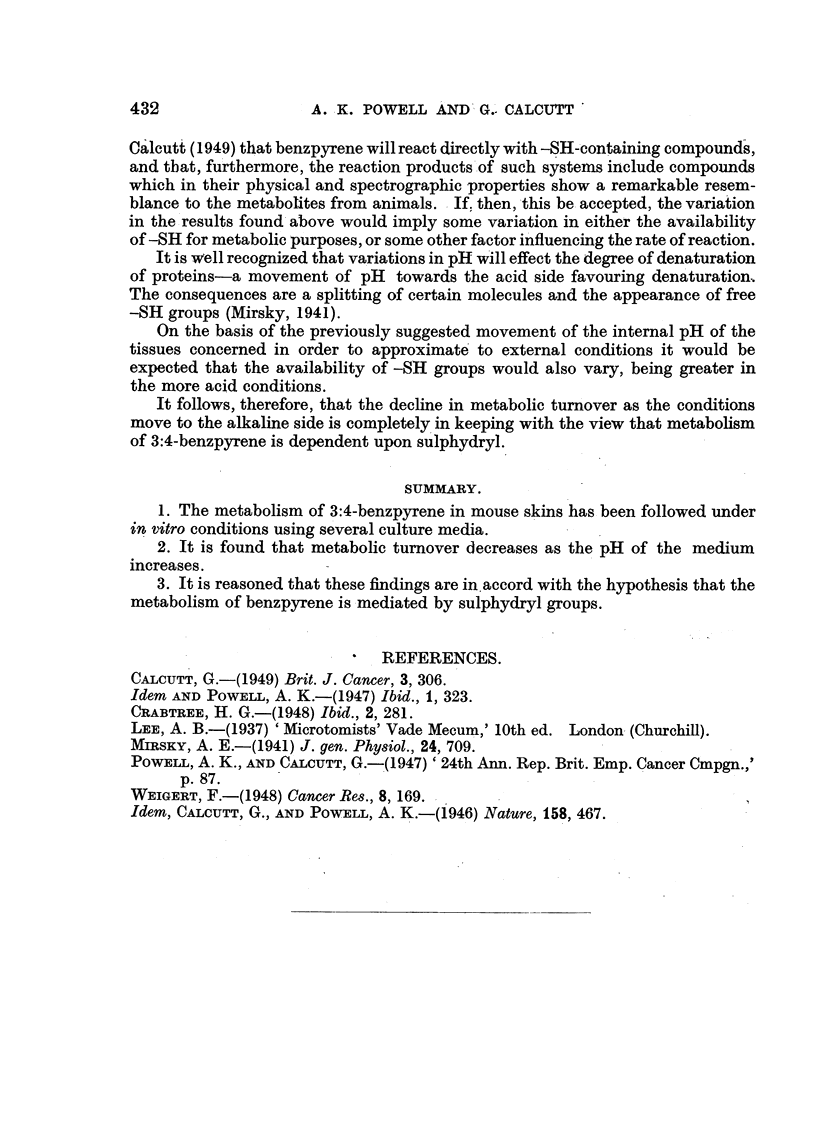# Further Experiments on the In Vitro Metabolism of 3:4-Benzpyrene in the Mouse Skin

**DOI:** 10.1038/bjc.1949.48

**Published:** 1949-09

**Authors:** A. K. Powell, G. Calcutt


### FURTHER EXPERIMENTS ON THE *IN VITRO* METABOLISM OF 3 : 4-BENZPYRENE IN THE MOUSE SKIN.

A. K. POWELL AND G. CALCUTT.

*From the Department of Cancer Research, Mount Vernon Hospital, and the Radium Institute, Northwood, Middlesex.*

Received for publication April 30, 1949.

A TECHNIQUE whereby the metabolism of 3 : 4-benzpyrene in mouse skin could be followed under *in vitro* conditions was described by Weigert, Calcutt and Powell (1946). The original experiments were carried out with Ringer Locke solution as the nutrient medium. Under these conditions measurable quantities of the metabolite $BpX_2$ were found, but in all cases the amounts concerned were very low. Since further alternative techniques failed to offer sufficient of the metabolite for chemical analysis (Powell and Calcutt, 1947), an attempt was made to improve the yield from the *in vitro* system by varying the nutrient medium. The results were a failure in this respect, and at the time no satisfactory explanation of them was available. However, in the light of more recent work—Calcutt (1949)—it is now possible to proffer a reasonable hypothesis.

### MATERIALS AND METHODS.

The skins used in these experiments were all taken from comparable positions on the flanks of 12-week-old male pure line Strong A mice. The technique for painting with the 0·3 per cent benzpyrene solution in acetone was that described by Calcutt and Powell (1947), and further treatment was as in earlier work (Weigert, Calcutt and Powell, 1946). Extraction and estimations of the metabolites were by the methods of Weigert (1948).

The nutrient media used were as below :

1. Ringer Locke Solution.
2. Tyrode Solution.
3. Hédon-Fleig Saline.
4. Pannett-Compton Saline.
5. Sterilized tap water.
6. Normal Saline.

All media were made up to formulae given in the 'Microtomists' Vade Mecum' (Lee, 1937).

In all experiments incubation was at 37° C. for a period of 8 hours.

### RESULTS.

Metabolism of the benzpyrene only occurred in the series on the four buffered media. In the case of the tap water and the normal saline the media were found to be turbid and the skins sodden and waterlogged. No indications of any change in the benzpyrene could be found in these cases, and it appeared that the conditions were too unfavourable for the survival of the skins. These have, therefore, been left out of further consideration.

With the buffered media metabolism took place and measurable quantities of $BpX_2$ were extracted. The results are shown diagrammatically in Fig. 1, the amounts of metabolites being estimated as μmg. of $BpX_2$. It is apparent that the metabolic turnover has shown a tendency to decrease as the pH of the medium has increased.

FIG. 1.—Metabolism of 0·3 mg. of 3:4-benzpyrene on 4 sq. cm. of mouse skin on various media.
(1) Ringer-Locke Solution –pH 7·3. (3) Tyrose Solution –pH 7·8.
(2) Pannett-Compton Saline –pH 7·5. (4) Hédon-Fleig Saline –pH 8·8.

### DISCUSSION.

As an attempt to produce benzpyrene metabolites in greater amount these experiments must be considered a failure. On the other hand, the variation in the yields obtained is in itself of some interest. Explanations of this based on the detailed composition of the media used appear to be of doubtful significance. The outstanding point seems to be the pH of the medium.

Under the conditions of experiment it is reasonable to assume that the tissues of the skins concerned became adjusted in some degree to the environmental conditions, that is, that the internal state of the cells approximated more nearly to the external pH than would normally be the case. The question arises, therefore, as to whether such a state of affairs could influence the metabolic turnover.

Taking advantage of some recent findings, it is believed that an explanation of the results can be offered along the above lines.

It has long been suspected that the process of carcinogenesis is intimately involved with sulphydryl groups. This view has recently been reinforced by Crabtree (1948), and the weight of evidence is now strongly in its favour. At the same time it has been suggested that the metabolism of benzpyrene is also mediated by –SH groups. This has received confirmation by the findings of

Calcutt (1949) that benzpyrene will react directly with –SH-containing compounds, and that, furthermore, the reaction products of such systems include compounds which in their physical and spectrographic properties show a remarkable resemblance to the metabolites from animals. If, then, this be accepted, the variation in the results found above would imply some variation in either the availability of –SH for metabolic purposes, or some other factor influencing the rate of reaction.

It is well recognized that variations in pH will effect the degree of denaturation of proteins—a movement of pH towards the acid side favouring denaturation. The consequences are a splitting of certain molecules and the appearance of free –SH groups (Mirsky, 1941).

On the basis of the previously suggested movement of the internal pH of the tissues concerned in order to approximate to external conditions it would be expected that the availability of –SH groups would also vary, being greater in the more acid conditions.

It follows, therefore, that the decline in metabolic turnover as the conditions move to the alkaline side is completely in keeping with the view that metabolism of 3:4-benzpyrene is dependent upon sulphydryl.

### SUMMARY.

1. The metabolism of 3:4-benzpyrene in mouse skins has been followed under *in vitro* conditions using several culture media.

2. It is found that metabolic turnover decreases as the pH of the medium increases.

3. It is reasoned that these findings are in accord with the hypothesis that the metabolism of benzpyrene is mediated by sulphydryl groups.

### REFERENCES.

Calcutt, G.—(1949) *Brit. J. Cancer*, **3**, 306.
*Idem* and Powell, A. K.—(1947) *Ibid.*, **1**, 323.
Crabtree, H. G.—(1948) *Ibid.*, **2**, 281.
Lee, A. B.—(1937) 'Microtomists' Vade Mecum,' 10th ed. London (Churchill).
Mirsky, A. E.—(1941) *J. gen. Physiol.*, **24**, 709.
Powell, A. K., and Calcutt, G.—(1947) '24th Ann. Rep. Brit. Emp. Cancer Cmpgn.,' p. 87.
Weigert, F.—(1948) *Cancer Res.*, **8**, 169.
*Idem*, Calcutt, G., and Powell, A. K.—(1946) *Nature*, **158**, 467.